# Visual hallucinations in psychosis: What do people actually see?

**DOI:** 10.1111/papt.12553

**Published:** 2024-11-18

**Authors:** Charlotte Aynsworth, Felicity Waite, Samuel Sargeant, Clara Humpston, Robert Dudley

**Affiliations:** ^1^ Cumbria, Northumberland, Tyne and Wear NHS Foundation Trust Newcastle upon Tyne UK; ^2^ Department of Psychology University of York York UK; ^3^ Department of Experimental Psychology University of Oxford Oxford UK; ^4^ Oxford Health NHS Foundation Trust Oxford UK; ^5^ Department of English Literature and Creative Writing The Open University Milton Keynes UK

**Keywords:** psychosis, qualitative analysis, reflexive thematic analysis, visions, visual hallucinations

## Abstract

**Background:**

One in three people with psychosis experience visions. However, little is known about what people see, and current treatments have limited benefits.

**Objectives:**

To improve the understanding and treatment of visions, this study explored the phenomenology of visions in people with psychosis.

**Methods:**

Twelve people with psychosis participated in semi‐structured interviews. Reflective thematic analysis was used.

**Results:**

Three main themes were generated covering important aspects of phenomenology: ‘Content’, ‘Coherence’ and ‘Quality’. The first theme ‘Content: People see people’, demonstrated that the most distressing visions were of people. The second theme ‘Coherence: Visions of people who behave like people’, captured how visions were coherent with real human behaviour, often by being multimodal experiences that spoke to and touched the observer. The third theme, ‘Quality: They look too real’ highlighted the compelling sense of authenticity of the visions, making them indistinguishable from reality.

**Conclusion:**

Visions represent what we expect to see in everyday life: people, who act and look real. This powerful combination provides insight into the absorbing and all‐encompassing nature of visions and their impact on participant's lives. The framework of ‘Content’, ‘Coherence’ and ‘Quality’ provides guidance to support clinicians and researchers to better explore the phenomenology of visions in psychosis.

## INTRODUCTION

Visual hallucinations, also known as visions, are the experience of seeing something in the absence of external stimuli (Waters et al., 2014). They are common in people with psychosis; the experience when people lose some contact with reality (National Institute of Mental Health, 2023). Approximately one in three people with first episode psychosis experience visions (Allen et al., 2023) and may be even more common in those who also report auditory hallucinations (Dudley, Watson et al., 2023). When present, visions are associated with significant clinical consequences, including more frequent and prolonged hospital admissions, greater likelihood of suicide (Baethge et al., 2005; Mueser et al., 1990; Oorschot et al., 2012), impaired functioning and greater symptom severity (Kreis et al., 2024). Yet currently, there is a lack of effective treatment for visions (Collerton et al., 2005; Thomson et al., 2017; Wilson et al., 2015). One route to produce more helpful psychological therapies is to first ensure a close and detailed understanding of the unique features and mechanisms that contribute to a specific problem (Clark, 2004). This approach has been successfully applied to other experiences reported by people with psychosis, for example persecutory delusions (Freeman, 2016; Freeman et al., 2021). However, such an approach is yet to be applied to visions.

To date, there has only been a limited number of studies exploring the phenomenology of visions, which have largely used quantitative measures. These found that typically people reported seeing humans, faces or figures with fewer seeing animals and objects (Dudley et al., 2012; Gauntlett‐Gilbert & Kuipers, 2005). This work also found that visions in psychosis are most likely to be multimodal experiences. Visions can also talk to and touch the person experiencing them (Dudley et al., 2018; Dudley, Watson et al., 2023; Hoffman & Varanko, 2006). Whilst informative, the quantitative measures commonly used in research often only ask a limited number of questions on visions (Aynsworth et al., 2017), which lack detailed exploration of these experiences, or include questions that are overly sensitive or too specific which can over‐ or under‐estimate people's experience of visions (Aynsworth et al., 2023). Thus, there is still a crucial lack of phenomenological description and understanding of visions.

Therefore, a necessary first step to improve the understanding and treatment of visions is to learn from first‐hand accounts of the experience. In this study, we used semi‐structured qualitative interviews to gather detailed descriptions of the phenomenology of visions. We wanted to address the research question: ‘What do people with psychosis who have visions actually see?’

## METHOD

### Design

A Big Q qualitative design was used (Kidder & Fine, 1987). A Lived Experience Advisory Panel (LEAP) helped to design the study and contributed to the interpretation and understanding of the findings. This study was approved by the NHS Health Research Authority (HRA) Newcastle and North Tyneside 1 Research Ethics Committee (reference: 21/NE/0099).

A critical realist ontological approach was taken to this research. This assumes that whilst there is one shared reality, peoples' perspectives of this are influenced by language, culture, and life experiences, meaning that the ‘true’ reality is only partially accessible (Clarke, 2021). A contextualist epistemology underpinned this position, which believes that humans act in context, and that they cannot be separated from the lives in which they live (Braun & Clarke, 2021). Within this stance, data collected does not represent a ‘true’ reality. Instead, researchers' subjectivity can be used to generate themes and understandings, which will be influenced by their own experiences and biases. Therefore, maintaining reflexivity is essential throughout the analysis.

### Participants

A criterion‐based purposeful approach was taken to form the participant group (Palinkas et al., 2015). This was based on the following inclusion criteria: (a) current experiences of visions defined as ‘visual perception‐like experiences that occur without an external stimulus. They are vivid and clear, with the full force and impact of normal perceptions, and not under voluntary control’ (American Psychiatric Association, 2013), (b) have a psychosis spectrum diagnosis (e.g. F20‐F29 as classified in the ICD‐10), (c) receiving care within an NHS mental health team, (d) aged 16 years or above and (e) willing and able to provide informed consent.

Exclusion criteria were: (a) visions only when under the influence of drugs or alcohol, (b) visions only when on the borders of sleep, i.e., hypnagogic/hypnopompic hallucinations, (c) visions that are only fleeting or rarely occur so a detailed interview would not be relevant, (d) significant brain injury or neurological condition, including dementia, to ensure that visions are unlikely to be a result of this, rather than psychosis or (e) moderate to severe learning disability meaning it could affect the capacity to provide informed consent.

Participants were recruited from NHS psychosis services in the North East of England.

### Procedure

The topic guide (Data S1) was developed in collaboration with people with lived experience, existing literature, and consultation with researchers in this field. The LEAP ensured the topic guide explored people's experiences of visions in an open and curious way. Whilst questions and prompts were included in the topic guide, it was used flexibly to respond spontaneously with questions or responses to each participant's narrative. Where people had more than one type of vision, the interview focused on the vision that they felt was most distressing or important to gain the most comprehensive understanding of their experiences.

Potential participants were initially approached by a member of their clinical team. Those interested were then contacted via phone to arrange a screening with a Clinical Psychologist, trained in qualitative research (CA). During which the Participant Information Sheet was shared and participants were given the opportunity to ask questions. If willing, an interview was then arranged and conducted by CA. Written informed consent was given prior to participation. This included consent to the use of pseudonymised quotes. Interviews were audio‐recorded on an encrypted dictaphone, and then transcribed verbatim and pseudonymised. Participants were able to choose their own pseudoynms.

### Reflexive thematic analysis

Braun and Clarke's six‐phases of analysis (Braun & Clarke, 2006) were used iteratively to ensure thorough data exploration. First, CA familiarised herself with the data, by transcribing the interviews, reading and re‐reading the data whilst making initial notes. Initial codes were then generated, by reviewing the data several times. This then allowed CA to generate themes, collating all data relating to each of them in Nvivo 12 (QSR International Pty Ltd, 2021). Themes were reviewed in supervision and with members of the LEAP, then re‐reviewed, generating several thematic maps to consider how best to convey the meaning of the data. Subsequently, themes were then defined, named, and ordered appropriately to reflect a narrative representative of participants experiences. An example of this iteration was that initially a theme of ‘Multimodal experiences’ was generated, which involved descriptions of the different multimodal (hallucinations occurring in different domains but seeming to originate from the same source) and multisensory hallucinations (hallucinations occurring in separate domains that were not from the same source; Toh et al., 2022). However, reporting in such a descriptive way missed the crucial element of how and why multimodal hallucinations particularly impacted on people and caused greater distress. Instead, on reviewing with the LEAP and supervisory team, what emerged was that what was most important in the accounts is that multimodal experiences were the most coherent with how real humans are experienced. We subsequently then removed the aspects of multisensory from the theme, as this told us less about the phenomenology of people's visual experiences and instead how they appraise their hallucinations more generally. Another example of the impact of the LEAP in analysis was the adaptation of Table 1. Initially, this had included participants demographics alongside a brief summary of what their visions were. However, this felt reductive of people's true experiences. Considering both participants and the LEAP members were passionate for people to truly understand their experiences, it was felt that using quotes as descriptions achieved this better as they captured much more detail of to what people's visions were really like. Given the descriptions used, we removed demographic information to help ensure their anonymity.

**TABLE 1:** Brief descriptions of participants visions.

Participant	Content of visions
Sarah	“I see [deceased friend]… I see her as a normal person…I seen her clear as day and I was hearing her clear as day, like she was sitting right beside us…When she talks to us, it's very, very negative. Telling us to hurt meself, telling us to do things…It's not like I can look through her, it's not an outline of a person. It's actually like I'm looking at you.”
Mick	“I'll have full on visions of people. I always see a man in the corner of me room. It was only ever when I was going to bed at night. Just literally like sought of stood there and stared. He's not like someone I recognise or anything like that. I can tell it's a man. He's quite big. You can see shoulders, y'know when you breathe and move up and down slightly. But no face, no hair, just like bald… it's as clear as you sat in front of me now.”
	“I was seeing people like walking through the garden, hiding, banging on the window, as if trying to like break in and smash the window or something like that… sometimes I can make out a face, sometimes it's like I almost recognise the person…other times there's like no face – I can see clearly they've got the marks of a face but there's no mouth, there's no eyes, it's just like holes.”
Emily	“I see mannequins. Sometimes they're dressed, sometimes they're naked. They're tall, discoloured like pale, sometimes they don't have facial features, sometimes they'll have facial features, like when I say facial features I mean like the eyes, the nose, everything you'd see on a human body I see there. It's a human face on a mannequin. There's no distinguishing sometimes if it's a male or a female. It's just a mannequin. They're always still in one position. Sometimes they're silent, some of them don't say anything, they just appear but then it's just distressing because they'll say things like, “Put cyanide in your mams tea, go on, do it, do it, hurt yourself”, which I try not to listen to. I can feel the breath in my inner ear. It's like somebody licking the insides of my ear.”
	“I see snakes. Big ones, erm, very long ones. I see them everywhere and they're just like crawling.”
Felix	“[I see] a man in a grey t shirt always walking past the window. All I can see is his blue pants and his grey t shirt and [he] kind of hasn't got a face. It's like a shadow, dark and black. It hasn't got any visual looks…. He just walks past slowly.”
	“Then I seen kids at the bottom of my bed… I used to hear, you know like when you can hear a school playground in the background, I was hearing that and obviously I was lying in bed and kind of waking up, they were there.”
	“I seen like a lot of shadows, like in the corner of me eye, like just kind of moving. But me most recent ones flies, and rats, and mice in me house.”
Scarlett	“[I see] things that I recognised and things that I'd never seen before like the children with no eyes or the black mass, kind of like a human but not. Just imagine like the darkest shadow you can have, the body's like an oval and it's got a round head and it's in the foetal position, but on all fours rather than at the side. It's like stretched like stretched out. It's like malformed but you can see the shape of the head, and then it can kinda like spread to try and feel the wall…But it's just so black…If I do choose not to ignore it, it normally brings on the tactile hallucinations where I feel itchy and like my skins on fire… I feel like there's ants crawling and it will get really hot and itchy and sometimes it feels like stuff behind my eyes as well. As if it's like almost forcing us to look at it.”
Tony	“Sometimes I see, like, figures, shadowy figures sometimes they're a little bit more fleshed out than that. Sometimes, I see deformed creatures, deformed babies, hybrid animals sometimes I see a mix between a man and a dog, which is quite negative and harmful and vicious, erm, other times I just see something – I just thought my [child] was there and [they] wasn't.”
	“I see this guy who looks like he's been burnt, he's a prominent one at the moment. He tends to hang about in me [child's] bedroom or the landing of the house. He doesn't speak but the voices I get will speak, are commenting on him. I never see his face ‘cause he's burnt there, so you see some eyes, he's got some black shit around his face, like black burns. He seems to come out of me kids wardrobe, he seems to live in there, he just walks past us, mainly at night, and he will just go to the other side of the room.”
Rodney	“The weird person I see is called [famous person]. He looks like him, I smell the smell [of rotten vegetables] before I see him and that's how I know when he's going to appear, ya kna. I just kna he's gonna bring us down and call us names ‘Ah you're useless, you should have done this, you should have done that.’”
	“I've seen dogs as well. I smell that first and then I can look down, and I can feel it moving around my legs, ya kna, woving in and out, what they do. And I think, ‘My god, who's dogs that?’”
Kyle	“I see evil clowns. They're opaque. So, it's exactly the same as me and you. I seen them [the clowns] in the street, but I mostly see them behind the windows. So, if I move back from the window, they've got their hands pressed on the window…They speak to me also… They tell me to kill people. Also calling me names, saying they're going to kill my family.”
Joseph	“[Famous person] drove past me. And then from that point onwards, I then began to see there was just like stream after stream after stream of famous people driving past me. Just anybody and everybody. It could have been, let's say about 200 hundred people driving past me… It's famous people and people connected to my life…. Some people drive past and I smile and give them a little joke and something, and some people would drive past and belittle [me]…It was all real to me at the time, they all seem real.”
Lily	“I see my [past abuser] obviously. He's dead now, but I see him… He started to threaten me, and he kept saying, ‘See I told you you'd never get rid of me’… he touches me whenever he goes passed, he will either pull my hair or run his hands down my face and my neck…He's just the same as like you and I talking. I can see him the way I can see you.”
	“I see dead people with half a face.”
Yogi	“All different kinds [of people] – mostly the military. I see kids, I see men, women, wounded people, upset people, happy people, it all depends. But most of them are the military who have been shot or even worse. Missing limbs. Blood all over their faces.”
	“[I also see] just random people that I don't know, I've never seen. They just stand there, staring at us either smiling, laughing. Or I'll have some ones that are crying. They'll have the facial expression, they'll point at ya, shake their head like you're doing something wrong.”
Liam	“It can vary. It can be completely different. Setting things alight in the house, when I've been sitting watching TV if I've had clothes drying on the radiator, me clothes will combust and go up in flames but the description of the actual flames are not real, they're almost cartoon‐like. The same car will go past, what 20 times but the best way of describing them is a cartoon style flat non‐ dimensional car, so it just looks like a cardboard cut‐out, so there's no shape or any sort of dimensions to it, it just a flat.”
	“There was a vision of me, of what I was wearing that afternoon, a vision of meself on fire. Flames coming off me back. The best way of describing it was a holographic type image, like a CCTV image of meself being replayed over and over, a loop on repeat, of me shouting…the image itself was that convincing, I think I convinced myself that it actually was happening, being visually replayed in front of us.”

### Reflexivity and considerations of data quality

Reflexivity “involves a disciplined practice of critically interrogating what we do, how and why we do it, and the impacts and influences of this on research.” (Braun & Clarke, 2021, p. 5). To conduct high‐quality analysis, it is essential to understand the researchers own perspectives (Elliott et al., 1999). To aid this, CA first engaged in a bracketing interview to help elicit values, assumptions, and biases prior to the interviews. This was supported by CA keeping a reflexive journal noting her reflections with regards to both conducting the interviews, and throughout the analytic process. To further reflect on subjectivity, coding and themes were regularly discussed and refined in supervision with FW and RD and with peer researcher SS, who has relevant lived experience, where different insights or perspectives were considered. The COREQ guidelines were also followed to support quality assurance (Tong et al., 2007; See Data S1).

In line with Braun and Clarke's (2021) ownership of subjectivity, the following statement is written in first person. I am a White British cis‐gendered female working as a Clinical Psychologist. I have specialised in working in a range of services delivering therapy for people with psychosis. This has influenced my position to believe that psychosis (and therefore visions) is largely impacted by people's life experiences and cultural context. Subsequently, I believe that peoples experience of psychosis and visions have important meaning for each individual and are not simply a symptom of a psychiatric disorder. The research team are experienced in developing and delivering psychological treatments for distressing psychotic experiences, with RD and myself specifically exploring visions within our research. Equally, SS along with lived experience also explores historical accounts of visual hallucinations from the medieval period onwards. Therefore, our knowledge will have influenced the current analysis (Braun & Clarke, 2021). However, we hope that the inbuilt quality considerations enabled us to recognise researcher subjectivity, whilst remaining open minded and curious to participants narratives and the subsequent data analysis presented below.

## ANALYSIS

### Contextualising the data

Thirteen people were approached for the study. One person declined to be screened. Twelve participants took part (8 male and 4 female; mean age 38.1 years, SD = 10.9 and range 22–58). Seven participants were from Early Intervention in Psychosis teams, four from Community Mental Health Teams, and one from an Inpatient ward. Most participants were diagnosed with Psychosis NOS (*n* = 8), three with schizophrenia and one with schizoaffective disorder. All but one of the participants were White British. Eight interviews were conducted at clinical bases and four at participant homes. Interviews were on average 81 min (SD = 13:58; range 57–101 min). See Table 1 for content of people's visions.

### Overview of analysis

Three main themes were generated and covered key phenomenological aspects of visions: content, coherence, and quality. The first theme ‘Content: People see people’ described how participants typically saw visions of humans or human‐like beings. This included two subthemes: (i) ‘Links with the past’ highlighted that visions can relate to participants earlier, often traumatic, life experiences in both direct and thematic ways and (ii) ‘Uncanny Valley’ identified that whilst visions typically took the form of someone with a human body, there was often a distinct feature or odd quality that identified the vision as not a real human. The second theme was ‘Coherence: Visions of people who behave like people’, which acknowledged that people's visions often shared dynamic human‐like qualities, which added to their compelling nature. This included a subtheme of ‘Coherence mirrors mood’ explaining how such coherency often fluctuated with participants moods. The final theme was ‘Quality: They look too real’. This captured the real‐life perceptual quality of the visions, and the implications of this.

### Theme 1 – ‘Content: People see people’

This theme describes the main content of the visions that participants reported seeing was people. Table 1 shows that a range of experiences were reported including animals, insects, or fleeting shadows, but the most distressing or important for participants was seeing people. These experiences seemed to be more distressing, and impactful on participant's lives, as they were much harder to ignore.

Some participants recognised the person they saw. This could be people from their past, famous people, or even people from their nightmares. Whilst for others, the people in the visions seemed unrelated and unknown.

He looks like [famous person] and he wears black clothes and all black jacket. He's all dressed in black…I just sit on the sofa and he stands up, he never sits down or anything. He just stands there and he paces. Rodney

Just random people that I don't know, I've never seen. I've never seen on movies, never seen on the telly. Just random people. They just stand there, staring at us either smiling, laughing. Yogi

The context in which people reported visions was important to the content. When visions of people were seen in participant's homes, they had developed into more of a ‘character’ or a personified entity with an energy or personality which could stay for prolonged periods of time. Such visions were in some way ‘fixed’ to a certain place (predominantly their homes), whereas in comparison when outside of their homes, the participants often experienced a random mix of unknown people, that were more fleeting.

Each person or whatever I see, it's like they're locked in the place that I see them. So ‘The Man’ is only ever in the bedroom. I don't see any of the others in the bedroom. All of the others will be in the garden. Mick

[Famous person] was at my last home I was in as well, but I never seen him out in the open or in the daytime or out walking, it's always in the house that I see [him]. Rodney

#### Subtheme 1 – ‘Links with the past’

This subtheme reflects how visions often resembled past, and often traumatic, life experiences. Despite not directly asking people about life experiences, traumatic experiences were often discussed. Participants spoke of having struggled throughout their lives. This related to visions in three ways. First, visions could be directly linked to past life experiences, which participants could often also recognise, such as seeing the vision of a person who was related to a traumatic event.

I see the older version of [past abuser]. The one I see more clearly is the older [version of abuser], but the younger [version of the abuser] is the one that strangles me. He's the one I see in the morning, with his hands around my throat. I can see the hate in his eyes, it's like he's soulless, there's no soul. Lily

Second, there were instances where the link was not obviously direct but rather there were thematic links between the visions in the present (such as how the visions made the participant feel) and how the participant felt during past traumatic life events. However, these links were not always obvious to the participant, but instead were more so to the researcher throughout the analysis.

Sometimes, like, at first my reaction was to like cowl and turn – not be like friendly, not to be accepting of the person driving past me – to see it as a humiliation… you know, like going back to the [birthplace] experience when I was very young, I was absorbing that material as a reaction to the people driving past me. …Yeh, it triggered me back to that kind of personality self that I've kind of brought in those early years. Joseph

Third, there could be an association between the time of adversity in participants lives and the visions in the present.

This was about the time I was getting bullied. I would have weird kind of nightmares. There used to be like a little like vent thing above the door…There were nothing there, I don't know why it was there, but I used to have nightmares about a faceless man looking through at us whilst I was sleeping and sometimes I'd wake up and I'd see the face there. Only for a second whilst, you know, your brain realises and your eyes realise that you're awake, but when I think about it, the kind of face there with just skin but like no, no eyes or mouth or anything like that, that is very similar to what I see [now]. It's almost like ‘The Man's’ a grown‐up version of that. Mick

#### Subtheme 2 – ‘Uncanny Valley’

The second subtheme relates to the ‘Uncanny Valley’ effect (Mori, 1970), which explains how objects that bear resemblance to humans, but lack fundamental human qualities, can provoke feelings of eeriness and repulsion. Whilst participants reported seeing humans, they often lacked specific features or had a distinguishable quality that highlighted that the vision was not a real human, which was often puzzling or unsettling for them. The missing human element varied, such as a bodily feature (often eyes or face) was missing or dismembered, the clothing worn was from a past era, or the vision embodied a caricature of a human such as clowns or mannequins.

I can tell it's a man. He's quite big. You can see everything…But no face, no hair, just like bald…No features whatsoever…It's as if I'm seeing three quarters of it and the rest hasn't loaded. Mick

These figures just had no face, flesh coloured, no clothes, no face, nothing identifiable as a human apart from the physique. Tony

They weren't like kids were dressed now, like. They were kind of dressed as World War 2 type people. So, like, they have faces and stuff, like little boys and girls and they were just playing around. They were dressed like old fashioned so I was kind of thinking ‘Why would they be dressed like that?!’ Felix

I see evil clowns. They've got big fuzzy orange hair. Quite plumpish. Snakes eyes, you know how it's horrible. In blue dungarees, face paint, red nose, sharp teeth. Sometimes, I'll see them walking down the street or hiding behind a bush, but mostly it's behind the window. Kyle

### Theme 2 – ‘Coherence: Visions of people who behave like people’

The second theme generated describes how the visions were very much like real humans owing to their human‐like qualities and the way in which they behave. This was often due to their multimodal nature, whereby peoples' visions often involved different sensory modalities, such as being able to touch or talk to the person, which added to the visions compelling realness.

He will pull at me, or I'll feel his hands around my neck, he touches me whenever he goes passed. He would just grab me, smack my face off the door. I felt his whole hand on my back push me down the stairs…He calls me every name under the sun. Lily

I would score the mannequins as being the most distressing because when they speak the like hairs on the back of my neck just stand up, it's like I've been electrocuted, it's like being fully wired to a telephone pole…It's the fact they speak. It's the fact I hear the voice and that's distressing. Emily

#### Subtheme 1: Coherency mirrors mood

This subtheme reflects how the level of coherence was often dynamic. It often varied depending on participants mood; with visions often becoming more coherent when low in mood. Several factors contributed to this: the multimodality and content of visions, their behaviour, and the duration they were present. When feeling low, the visions were more likely to be multimodal, such as the visions were more likely to speak and touch the participants. The content could also change when low in mood, with the visions saying more derogatory or insulting comments in a more aggressive nature to the participants, such as by shouting at them. The visions behaviour would also appear more negative and aggressive such as intruding into peoples' personal space, grabbing the participant, or staring in an intimidating way. And finally, when participants' mood was low, the visions would stay present for longer periods of time. Such factors added to the coherent nature of visions making them more believable. In comparison, when feeling calmer, participants experienced the visions as less multimodal, so the visions would speak less or not at all, and their behaviour was less aggressive. Participants found this less distressing, therefore, enabling them to manage their visions more effectively.

Well, sometimes she talks to us when she's there, when I can see her…when she talks it's very negative. A lot of the time it's when I'm really, really low and I'm feeling really low about meself. Sarah

### Theme 3 – ‘Quality: They look too real’

The final theme generated was in relation to the perceptual quality of how the visions appeared to participants. Visions were not fleeting images in the corner of their eye or vague shadows. Instead, they had a compelling sense of reality in that visions shared the same level of perceptual detail, clarity, intricacy and depth as real people or objects. Such complexity and vividness of the visions, combined with the coherency of these, seemed to make the visions all‐absorbing, whereby participants reported finding it almost impossible to disengage their attention.

These visions have always been very real to me, they're as real as you to me sitting [there], I could be touching you and it would feel like I'm touching a human, so they're not something like smoke when you put your hand through it, it disappears. They're very physical and they're very real. Emily

[When paying them attention] they become more real. They're real already but they become more outlined. Like my whole attentions on them, they seem like everything's going blurry but them [the visions]. It's like when my whole attentions on them, nothing else matters ‘cause all my attention goes on them. ‘Cause I want to know if they're actually real or not. Yogi

It's like watching a car crash, d'ya know what I mean? You can't look anywhere else. You just like absolutely locked onto it, like. You feel like you wanna run away and hide from it. But you can't take your eyes off it. Mick

Consequently, owing to such difficulty discerning if visions were real or not, participants struggled to trust their own eyes. Understandably this had a detrimental impact on participant's lives, with them discussing being unable to leave their homes owing to a fear of being overwhelmed, acting inappropriately or being judged by others.

Yogi “That's why I don't like being around people, I don't like going out anymore ‘cause I don't know who's real and who's not. If I go out – me mate normally says ‘Are you coming to [restaurant] or a drink at the pub?’, I'll just back down straight ‘No’. ‘Cause the last time I went in, I saw visions all over the place.”

Interviewer “What happened then?”

Yogi “I had to go home. In a bar full of people. Real people and you're seeing even more people that aren't even there. Then other people start looking at you going, ‘What's he looking at?’. Just doesn't feel right.”

I get really paranoid that people are staring at us and because I don't want them to. I don't know if it's going to be real or not like cause the man I saw, who [partner] didn't see. I don't know if he was just a man that left [the area] or if it was someone I was just seeing. Because [partner] should've seen him by the direction of where he was looking, so I don't like it when that happens so I try and avoid it. I don't want to go out and have a conversation with someone and my partner goes, ‘Who are you talking to?’ Scarlett

## DISCUSSION

To our knowledge, this is the first qualitative analysis exploring the phenomenology of visions in people with psychosis. Twelve participants provided rich accounts of their visions, demonstrating the incredible intricacy and intensity of these experiences. The three themes generated highlighted the content, coherence, and quality of visions. Typically, visions represented what people would expect to see in the everyday world: people, who behaved like people (such as having the capability to talk to and touch participants, increasingly when participants were in lower moods as if mirroring their feelings) and looked like humans (shared the same perceptual qualities as real living people). This powerful combination of content, coherence, and quality provides an important insight into how the complexity and vividness of visions contribute to their compelling sense of reality.

These findings are consistent with previous research which found that participants most commonly see people (Dudley et al., 2012; Gauntlett‐Gilbert & Kuipers, 2005), and that these are often multi‐sensory and or multi‐modal experiences (Dudley et al., 2018; Dudley, Watson et al., 2023; Hoffman & Varanko, 2006; Toh et al., 2022). Yet our study provides much more detailed descriptions of precisely what people see, how the vision behaves and how they appear to the observer. Our findings support the notion that visions are seemingly much more complex, multi‐modal experiences that can interact with people. They are not silent or static images that can be easily dismissed. Such findings provide insight into why visions can be so distressing and impactful on people's lives (Baethge et al., 2005; Chouinard et al., 2019; Mueser et al., 1990; Oorschot et al., 2012).

Furthermore, whilst not specifically asked about, we found that participants spontaneously spoke about how their visions were related to important (often traumatic) experiences in their lives. As with auditory hallucinations (Hardy et al., 2005), visions seem to have direct content and thematic linkage with adversity and meaningful events. Emphasising that the content or experience of visions is meaningful in the context of the person's life.

In terms of theoretical understanding, the Predictive Processing Framework (PPF; Friston, 2009) could explain seeing visions that represent what we might expect to see in our social world. PPF suggests that perception is an unconscious process which combines sensory data from the environment with prior beliefs (or expectations) about the world. Therefore, hallucinations may arise by an “over‐weighting of prior beliefs relative to sensory perceptions” (Kafadar et al., 2020). As social creatures, we are primed to see dynamic people; static/silent humans are less usual, which could lead the brain's expectations to override perception to make the visions more multimodal in content. The relationship of visions to past trauma and the increasing levels of coherency when people were low in mood (as if the visions were mirroring their moods) could also be explained by this theory (Lyndon & Corlett, 2020) – whereby difficult life experiences (both day to day and past) influence people's expectations and biases to an extent which are difficult to undo. Hence such prior beliefs can override, or even ignore, other sensory data and lead people to see more distressing negative content or people from their past trauma in their visions. However, discussions with the LEAP questioned the relatability of the PPF to explain visions. Whilst there was an acknowledgement that predictive processing likely plays a function in all of our perceptions and can lead to misperceptions at times, it lacks sufficient detail to provide explanations as to how peoples visions could be so vivid, complex, and prolonged in duration. In the absence of acceptable or convincing explanations, patients with psychosis have described engaging in a process of meaning making (Aynsworth et al., in prep). From a cognitive perspective, appraisals are central to determining the impact of experiences (Beck, 1976). Working with appraisals is a key part of cognitive therapy approaches to voices (Morrison, 1998) and visions (Collerton et al., 2005; Thomson et al., 2017; Wilson et al., 2015). Therefore, there is an opportunity for further development of cognitive approaches to visions drawing on the phenomenology outlined here to help make sense of (or appraise) people's experiences. For example, seeing visions of people that look and behave like real people (i.e. the coherence and quality of the phenomenon) could provide insight as to why people may appraise their experiences as real people and are therefore difficult to dismiss. Further to this, an interaction between mood and the phenomenology of visions was described: when participants were lower in mood, visions would present as more negative or aggressive in their behaviour, which could offer an understanding as to why visions are often appraised as threatening (Dudley et al., 2012; Gauntlett‐Gilbert & Kuipers, 2005).

### Limitations

There are several limitations to the study that need to be considered. First, the sample was predominantly White British which reflects the demographics of the North‐East of England where the study was conducted (94% White British according to the latest census; Office for National Statistics, 2022) and who access local mental health services (83.7% White British; Campbell‐Lee & Cohen‐Tovee, 2023). However, given the findings relating to past experiences and social context influencing the content of visions, we expect there may be differences with a more diverse participant group, especially given the inequalities and discrimination that different ethnic groups can face (Equality and Human Rights Commission, 2016; Runnymede Trust, 2021). This is of particular importance given the overrepresentation of people with psychosis from ethnic minority groups (Baker et al., 2021; Selten et al., 2020). Second, this study focused discussions on the most important or distressing visions that people wanted to talk about. However, many people do not find visions problematic (Aynsworth et al., 2023; Linszen et al., 2022). Therefore, such findings may not apply to those who are less distressed by their visions, for example people in the earlier stages of psychosis (Dudley, Denton et al., 2023) or older adults where visions are typically unimodal (Dudley et al., 2018). However, the limited work to date has shown similarities between non‐clinical and clinical groups in terms of content of what is seen (people, faces). Third, this study explored visions in people who were able to provide rich detailed accounts of their visions. A greater range of phenomenology may be experienced which has not been captured here.

Future studies investigating different populations and experiences would be helpful to extend our understanding of visions. For example, phenomenology of visions may differ between people diagnosed with psychosis and people diagnosed with other conditions such as eye disease or dementia, where there is an indication that visual hallucinations may be more unimodal (Dudley et al., 2019). Alternatively, given our results are consistent with the work indicating an important role for trauma in psychosis (Dudley, Turkington et al., 2023; Shevlin et al., 2007; Varese et al., 2012), especially in those with visions (Solesvik et al., 2016), it would be helpful to consider if different types of trauma may lead to different phenomenology of visions.

### Research and clinical implications

The findings provide a framework to guide both clinical and research questions through three important areas: the content, coherence, and quality of visions. First, to ensure visions are accurately assessed, better assessment tools, including psychometrically robust measures, are required. This framework could also guide questions during clinical assessments. First, clinicians could explore the detailed content of what people see. This could identify links to past trauma or identify any unique qualities of the visions which could identify this as a vision compared to a real person (such as through the Uncanny Valley effect). Second, clinicians could consider the coherency of their visions such as exploring multimodal aspects of these, and how the visions can change depending on a person's mood. Third, the quality of experiences could be explored to gain more understanding of how the visions physically appear to the person and how that may impact people's reactions to this. By capturing more comprehensive information about visions, it could support the delivery of core therapeutic principles in clinical care, such as normalising and empathy to reduce internalised stigma (Aynsworth et al., in prep; Renouf et al., 2018) and enhance therapeutic alliance (Goldsmith et al., 2015). More specifically, targeted techniques could be developed from this learning. We noted the ‘Uncanny Valley’ observation that visions often lacked a human quality, which people found distressing. However, such discrepancies could in turn be used to generate doubt and help to test if the vision is real or not, which could foster more helpful coping. E.g. if the vision is missing eyes or a mouth, then it could be used to consider the coherency of the experience. This could be a route to help meet the need for acceptable and convincing explanations of visions that patients are seeking (Aynsworth et al., in prep).

## CONCLUSION

Traditionally visions have been viewed as fleeting, unimodal experiences, which lack perceptual clarity. However, the first‐person accounts in this study indicate that visions are much more complex, vivid, and absorbing experiences which share the same perceptual qualities as other humans or objects in the everyday world. We provide a framework to support clinicians and researchers to ask about people's visions: to explore the content of what people see, enquire about the coherence of the visions, and consider the perceptual qualities of these.

## AUTHOR CONTRIBUTIONS

**Charlotte Aynsworth:** Conceptualization; data curation; methodology; formal analysis; project administration; writing – original draft; writing – review and editing; visualization; resources. **Felicity Waite:** Conceptualization; methodology; validation; supervision; resources; visualization; writing – review and editing. **Samuel Sargeant:** Conceptualization; methodology; resources; writing – review and editing. **Clara Humpston:** Writing – review and editing. **Robert Dudley:** Conceptualization; methodology; resources; supervision; validation; visualization; writing – review and editing.

## CONFLICT OF INTEREST STATEMENT

R.D. declares royalties received for workshops on the topic of visual hallucinations and their treatment. C.A., F.W., S.S. and C.H. have no conflicts of interest to declare.

## Supporting information


Data S1:

## Data Availability

The data will not be made available as they contain highly personal, and potentially identifiable information and the right to share this information was not part of the consent procedures.
