# Supplementary material for: Visual hallucinations in psychosis: What do people actually see?
Source: Psychol Psychother. 2024 Nov 18;98(1):58–73. doi: 10.1111/papt.12553 (PMC11823304; doi:10.1111/papt.12553)
Supplement: Supplementary file 1 — Data S1: [file PAPT-98-58-s001.zip › Phenomenology Supplementary material A.docx]

Visual hallucinations in psychosis

Qualitative Interviews

Topic Guide

**Introduction**

Visual hallucinations (VH), also known as visions, occur when peoples see things that are not actually there. Eyes can often play tricks on people, leading them to see something that isn’t there but for most people this is a fleeting and an easily understood experience, but this seems to be more frequent and upsetting for people with psychosis, where they occur in 27% of people. The occurrence of visions is often associated with more frequent and prolonged hospital admissions (Baegthe et al., 2015; Meuser, Bellack & Brady, 1990) and poorer treatment outcomes (David et al., 2011; Mueser et al, 1990; Oorschot et al., 2012). Despite such negative impact of visions, relatively little is known about these. What is known has largely been explored using quantitative measures, which have questionable reliability and validity (Aynsworth, Collerton & Dudley, 2017).

The aim of these interviews is to be the first to provide detailed first person perspective experiences of visions in psychosis. By carrying out semi-structured interviews, designed collaboratively with people with lived experience, we aim to understand more about the content of what people see, to try to identify important maintenance factors of these experiences, and better understand the impact of visions on people’s lives and clarify their treatment preference for visions. From this, we hope to develop more effective and targeted treatments for those who experience visions.

**Overview of interview**

1. Introduction to topic of VH
2. Content of VH
3. Sense making
4. Emotional reactions
5. Beliefs about visions
6. Behavioural reactions
7. Impact
8. Stigma
9. Treatment

Initially the interview start with introductions from the interviewer, explaining their role and how the interview will be carried out. The interview will start with an open question to elicit a narrative context for people’s visions. This will allow the interviewee opportunity to feel more relaxed with the interview, having more influence on what is being shared and how, allowing both parties to build rapport. The interviewer will then use their answer as a basis to explore further other areas of their experiences to elicit details on the content, maintenance, impact of the VH and treatments. The interviewer will respond to their narrative in a flexible and curious way to naturally encourage conversations with the participant. Prompts are included so that the interviewer can invite further detail and explore the participants experiences in more detail. The interviewer will hold an open, curious, validating and non-judgemental stance throughout the interview to enable the participant to feel safe and supported.

**Interview Schedule**

These are the types of questions and topics to be covered during the interview; however, the questions will evolve in response to the participant’s language and understanding. It is hoped that the questions will act as prompts to encourage the participants to speak freely and more detailed about their experiences. Questions can be rephrased to ensure participants understanding and the order is flexible to respond to the participant’s narrative. The interviewer will use the participants own vocabulary to enhance the discussions of their experiences.

*General introductions – scene setting*

- Thank you for being to be part of this interview.
- Researcher introduce themselves and role
- Lasts about 60-90 minutes
- No right or wrong answer
- Reflective discussion
- Don’t have to answer
- Can take a break at any time

Before we begin, can I check you are in a comfortable place to discuss your experiences? In which you can speak openly, with minimal distractions?

*Collect oral consent, if not conducted face to face.*

If you’re happy then I will start the audio recording and ask you to confirm again that you are happy to take part in this interview. I will read through a series of questions to confirm you are happy with all aspects of the study.

- START RECORIDNG -

COMPLETE ORAL CONSENT FORM

- END RECORDING -

I will now start recording (again) and we can get started.

- START RECORDING -

I know I have just talked this through but just to confirm for this recording – are you happy to take part in this interview?

1. **Introduction to the topic of VH**

**To begin with, you were referred by your care team. Do you mind just explaining a little more about your involvement with care team such as who you work with and what they offer you.**

***The purpose here is to encourage the person to talk about a topic that they may be more familiar discussing. It will also foster rapport allowing the interviewer to understand the participant’s position in relation to services.***

**Brilliant, thank you. We have discussed that the main focus of this topic is VH. What do you refer to your experiences as such as VH or vision?**

- *Use the participants term of reference for the interview.*

**I’d like to begin by hearing more about your experiences of VH (or other preferred term)?**

*Prompts:
- How often do you experience these?*

- *What sorts of things do you see?
  - When did you first experience these?
  - When did you last experience one of these?*

1. **Content of vision**

**I would really like to imagine what it looks like when you see them so I can try to understand better what it’s like for you, can you describe this to me in more detail?**

*Prompts:*

*-Option to draw their VH, if they wished to.
- How does it appear? Is it as in normal perception or does it have different qualities?
- Is it clear? Vivid? Bright? Blurred? Colour?*

*- What size is it? Normal size in reality or does it differ to the size which you’d expect it to be?*

*- How long does it last for? Does it usually last this amount of time or does it vary?*

*- are there any patterns to when you see it? E.g. time of day or setting.
- Does it feel real to you? During and after. What makes it so?
- How does the vision present itself to you? What do you notice something first?
- Do you recognise the vision?*

*- Are visions worse or change at all depending on how you are feeling? E.g. when you feel more unwell.*

*- Have you ever experienced something disappearing from your eye sight? Something that was there that suddenly was not anymore?*

*- Does vision change in different situations e.g. busy crowded places compared to when alone*

*-Do you experience the visions in the same place? Or have you noticed a pattern to when they might appear to you?*

*- Does the vision ever talk to you? If so, what does it say? Is it present when it does? Is the movement congruent as if the vision is talking? Or do you ever heard the voice of vision when you can’t see it? How often is this the case?
- Does it ever touch you? Can you tell me more about that? Are they times when it has touched you but without you seeing the vision at the same time? How often is this the case?
- Does it ever have a smell associated with it? Before or after. Can you tell me more about that?*

*- If you experience hallucinations across different senses, do you think they are related to each other?*

*-Sometimes visions can portray everyday events such as a woman talking, sometimes that can be more supernatural or unusual such as a dog talking to you. What are yours like?
- How does it end? When do you know that it’s gone?*

*- What is the worst bit about the vision?*

*- What is the best bit about the vision?*

**3. Sense-making – understanding of visions

Why do you think when you see visions? What do your visions mean to you?**

*Prompts:*

- *How you do make sense of the vision/s?*
- *Do you have a theory as to why you see visions?*
- *What do you think keeps your vision appearing? What leads you to keep seeing visions?*
- *Do you feel it’s something related to you or something external – out of your control or influence?*
- *Do you think the visions are related to something personal or something that has happened in your life?*

1. **Emotional reactions**

**You’ve described seeing X. How does it make you feel when you see it?**

*Prompts:*

- *What is it that makes you feel this way? Can you tell me more about that?*
- *Is it related to the vision (e.g content, presence), yourself (controllability, stigma) or something different?*
- *How do you feel when you see it?*
- *How do you feel when it’s gone?*
- *How would you like to feel when you see it?*
- *Does it make you feel differently at different times? If so, can you tell me more about that?*
- *Has your emotional reaction changed during the course of your seeing the vision?*
- *How do you feel now talking about it?*
- *How strongly do you feel that emotion?*
- *Where do you feel that in your body?*
- *What is the worst moment, and what do you feel right then?*

1. **Beliefs about visions**

***What do you believe is happening when you see the vision - during and after?***

*Prompts:*

- *When you see X, what do you think to yourself?*
- *What do you think about the vision?*
- *What do you think about the vision when it’s not there?*
- *What do you think about yourself when you see it?*
- *How do you feel about seeing visions?*
- *Do you feel that you have any control or power over the vision?*
- *What does it make you believe about yourself?*
- *What does it make you believe about the world?*
- *What would you think of someone else who reported seeing things?*
- *Does it cross your mind that you may be at risk of being hurt by the vision, or that you may be losing your mind?*
- *Does your beliefs change across time e.g when you are in a crisis compared to when you are not? Does it fluctuate with how you are feeling?*
- *Are there any other places or images that are similar to your visions? E.G have you ever seen your vision in something else e.g. a tv show or film or something similar to your vision?*

1. **Behavioural reactions**

**You described seeing X, what do you do when you see that? E.g. how do you react or behave?**

*Prompts:*

- *What do you do to cope? What do you do to keep yourself safe?*
- *What makes you behave in that way? What drives that behaviour?*
- *What are the consequences of you behaving that way?*
- *Have you ever behaved differently? If so, what happened?*
- *What would happen if you didn’t behave like that?*
- *Is this how you would like to behave when you experience this?*
- *Are there particular behaviours that are helpful or unhelpful for you?*
- *Has anyone ever told/helped you with how to respond or what to do when you see the vision?*
- *(link back to the appraisals and ask if this stops them ‘losing control’ or from their appraisal coming true…)*

1. **Impact**

**You’ve described really well what you see and how it impacts you in the moment, does seeing visions have an impact on your life more generally? E.g. does it impact on your ability to do things that you want to do in your life or affect your relationships?**

*Prompts:*

- *Do the visions help you to do things?*
- *Do the visions have a helpful impact on your life in anyway?*
- *Does seeing visions impact on your relationships? If so, can you tell me more about that?*
- *Would your life be different without visions? If so, in what way?*
- *How has life changed for you since you started seeing visions?*
- *Has seeing visions impacted on your work life?*
- *Has seeing visions impacted on different areas of your life?*
- *Has visions changed your ability to go to different places e.g crowded or busier places?*

1. **Stigma**

**Do you think there is a stigma attached to you experiencing visions?**

*Prompts:*

- *Has seeing visions impacted on how you view yourself?*
- *Has seeing visions impacted on how others view you? Public, closer friends and family.*
- *How have people reacted when you tell them about seeing visions? Have you ever experienced positive or negative reactions to your visions?*
- *If you have not told anybody, what stops you from sharing this? Would you like to be able to share this?*
- *Do you know of other people that see visions?*
- *How do you think people view people who see visions?*
- *Are you more awareness of stigma differ across situations?*
- *How do you feel about the stigma?*

1. **Treatment**

**Have you ever been offered or received treatment for the visions?**

*Prompts:*

- *If so, can you tell me more about that? What was it? Did it help you?*
- *If not, how does that make you feel? How do you make sense of not being offered a treatment for these?*
- *Would you like a choice in the treatment to help you? If so, what types of treatment would be helpful?*
- *Do you want to continue seeing visions? Do you consider them a part of you?*
- *In an ideal world, what would be the outcome of your life and the visions? Would they stay or go?*
- *What do you think could make a difference to this?*
- *If available, would you like an intervention to help you to understand and manage visions?*
- *If you could have a treatment, what would it look like? What would it involve?*
- *In comparison to other difficulties that you may experience such as voices, anxiety etc, where does a treatment for visions fit in a ‘priority list’?*
- *If not a priority, what else do you feel needs treatment or resolving before focusing on visions?*
- *What places visions in that position on your ‘priority list’?*
- *Is there anything less of a priority for treatment compared to visions?*
- *If I could tell you that you could live a life without visions, what would you say? How would you feel?*
- *If I told you that you could live a life with visions but be less distressed by these, what would you say? How would that you make you feel?*

**Is there anything that we’ve not asked about that you want to share, or think would be important for us to know to understand your views?**

**We’ve reached the end of the interview. Thank you for your time today, we really appreciate you sharing your experiences with us. We will be in touch with the results of the study, if you would like to find out more about this.**

--END RECORDING –

*General prompts:*

- Can you tell more about that?
- How does that make you feel?
- Are there any times where you’ve felt or behave differently?
- What are the clues that tell you that?
- What makes you think that?
- What else have you thought about that?
- Has anything else come to mind when you experience that?
- Acknowledge the participant is separate to their experiences.
